# Utility and safety of a self-developed patent chest strap in endoscopic transaxillary thyroid surgery: a prospective comparative effectiveness study

**DOI:** 10.1038/s41598-026-64452-5

**Published:** 2026-07-27

**Authors:** Jie Shang, Yi’ning Wang, Xiwei Jiang, Jinqing Zhang, Xiaoyan Zou, Ying Xu

**Affiliations:** 1 Department of Nurse Center, Shandong Provincial Third Hospital, Shandong University, Jinan City, China; 2 Department of Breast and Thyroid Surgery, Shandong Provincial Third Hospital, Shandong University, No 12 Wuyingshan Middle Road, Tianqiao District, Jinan City, 250031 Shandong Province China; 3 Department of General Surgery, Peking University Medical Lu Zhong Medical Center - Lu Zhong Outpatient Department, No.65. Taigong Road, Linzi Area, Zibo City, Shandong Province China

**Keywords:** Endoscopic transaxillary thyroid surgery, The patent chest-strap, Traditional bandages, Phaseⅰreal-world study, Utility and safety, Diseases, Health care, Medical research

## Abstract

A novel patent chest-strap was developed for use in endoscopic transaxillary thyroid surgery. We conducted a phase prospective study in China to evaluate its utility and safety. This phase 1 study evaluates the utility, safety, and effectiveness of a novel patent chest-strap in a real-world clinical patient population. This single-center, open-label, prospective, randomized study was conducted from July 1, 2025, to March 31, 2026, to evaluate the long-term safety and efficacy of a patented chest strap (PCS) in patients undergoing endoscopic transaxillary thyroidectomy. Patients older than 18 years who underwent the procedure with or without the chest strap were enrolled. The primary endpoints included postoperative drainage outcomes and total usage of the PCS, which served as measures of effectiveness. Secondary endpoints focused on strap-related complaints, assessed in comparison with traditional bandages (TB), to evaluate safety. A total of 108 patients were included in the PCS group and 126 in the TB group. In the PCS group, the mean age was 43.5 ± 16.2 years, and 61 patients (56.48%) were female. Compared with the TB group, the PCS group showed statistically significant differences in height (1.67 ± 0.03 m vs. 1.73 ± 0.04 m; *P* < 0.00001), weight, and body mass index (*P* < 0.0001). The mean postoperative drainage volume on the first day was 50 mL in the PCS group and 48.5 ± 9.19 mL in the TB group. Drainage volumes differed significantly between groups on the second day (36 ± 8.49 mL vs. 44 ± 12.73 mL; *P* < 0.001) and the third day (35 ± 7.07 mL vs. 25 mL; *P* < 0.001). The total postoperative drainage volume was lower in the PCS group than in the TB group (111.92 ± 13.44 mL vs. 122.5 ± 24.75 mL). Complications were more frequent in the TB group. Skin allergy occurred in 40 patients (31.75%) in the TB group compared with 9 patients (8.33%) in the PCS group. The patent chest-strap has the postoperative benefits for patients with endoscopic transanxillary thyroidectomy, and suffered fewer ratio of complications. This phase real-world study performed a positive feedback for a self-developed clinical patent transformation achievement in China.

*Registration*: Chinese Clinical Trial Registry (https://www.medicalresearch.org.cn/) ChiCTR2500107867 (Registered data: 15 August, 2025)

**Supplementary Information:**

The online version contains supplementary material available at https://doi.org/10.1038/s41598-026-64452-5.

## Introduction

Endoscopic thyroidectomy via the axillary approach has emerged as a significant advancement in minimally invasive thyroid surgery over the past two decades^1–4^. Initially developed to address cosmetic concerns by avoiding a visible cervical scar, this technique utilizes an incision in the axillary to create a working space and access the thyroid gland^5–7^. Refinements in surgical instrumentation, such as high-definition endoscopes and ultrasonic energy devices, coupled with accumulated surgical experience, have progressively expanded its indications and improved its safety profile. It is now a well-established option for selected patients with benign thyroid nodules, low-risk papillary thyroid carcinomas, and Graves’ disease, particularly for those who prioritize cosmetic outcomes. Compared to conventional open thyroidectomy, the transaxillary approach offers superior cosmetic satisfaction and may be associated with reduced postoperative anterior neck discomfort and paresthesia^8–11^.

However, the unique surgical trajectory and manipulation through a remote incision create specific postoperative requirements. Effective wound management and compression at the chest wall incision site are crucial to minimize seroma formation, hematoma, and ensure optimal healing^12^. Currently, postoperative compression typically relies on generic elastic bandages or straps applied across the chest. These traditional bandages (TB) are not specifically designed for the biomechanics and anatomy following axillary-approach surgery. They often provide sub-optimal pressure distribution, leading to issues such as slippage, uneven compression, and patient discomfort, including skin irritation, pressure sores, and restricted respiratory movement. This mismatch between a specialized surgical technique and a non-specialized postoperative support device represents a notable gap in perioperative care.

Despite the widespread adoption and standardization of the endoscopic transaxillary thyroidectomy technique, a dedicated postoperative compression device specifically engineered for its unique requirements has been lacking. To address this gap, our team developed a novel chest strap, which received authorization as a Chinese utility model patent in October 2024 (Patent No.: ZL202323164047.5). This present study represents the first-phase, real-world clinical evaluation of this patented chest strap (PCS). It was conducted to assess the initial safety and effectiveness profile of the device in patients undergoing this surgical procedure, providing preliminary evidence for its potential to improve postoperative outcomes.

## Methods

### Study design

This single-center, open-label, prospective, randomized study (Chinese Clinical Trial Registry (https://www.medicalresearch.org.cn/) ChiCTR2500107867) conducted from July 1, 2025, to March 31, 2026. The study was approved by the Human Ethics committee of the Shandong provincial third hospital (No. KYLL-2025140). Every human participant have provided their consent in line with the Declaration of Helsinki.

### Patient selection and intervention

**Fig. 1:**
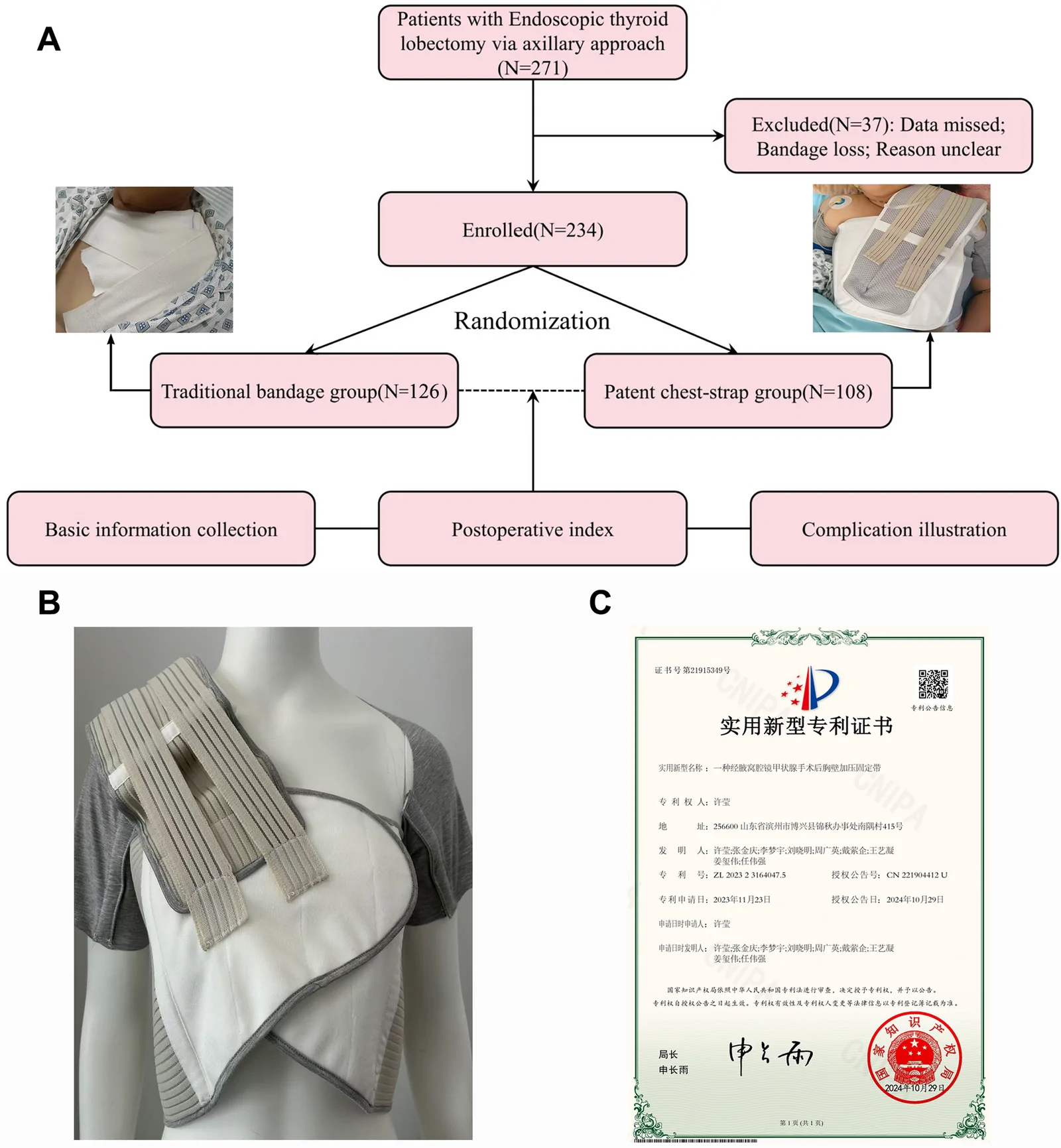
Flowchart of patients’ inclusion and Patent verification.

From July 1, 2025, to April 30, 2026, more than 500 patients underwent thyroid surgery for benign or malignant tumors at the Department of Breast and Thyroid Surgery, Shandong Provincial Third Hospital. From this cohort, 308 consecutive patients who underwent endoscopic transaxillary thyroidectomy were initially enrolled. These patients were randomized into two groups for postoperative wound management using a computer-generated simple random sequence (without stratification). Allocation concealment was ensured by sequentially numbered, sealed, opaque envelopes, prepared by an independent researcher not involved in patient care. At the end of surgery, patients received either the novel patented chest-strap or traditional bandages. PCS was available in three sizes (M, L, XL) based on individual chest circumference to ensure a tailored fit. TB consisted of a thick gauze pad secured with a circumferential elastomeric adhesive tape. Patients were excluded from the final analysis for the following pre-specified reasons: missing key perioperative data, accidental removal or loss of the dressing prior to scheduled drainage tube removal, and any other protocol deviations that could confound the outcome assessment. After applying these criteria, 108 patients remained in the PCS group and 126 in the TB group (a total of 234 patients from the original 308, due to a 24.0% exclusion rate) (Fig. 1).

### Effectiveness assessments

The efficacy analysis population comprised patients who received the allocated intervention and had complete data for the pre-specified effectiveness endpoints. For this population, baseline demographic and clinical characteristics, including sex, age, height, weight, and body mass index (BMI), were collected. The primary effectiveness endpoint was postoperative drainage volume, with all output recorded meticulously each day prior to surgical drain removal. The drainage metrics assessed included the daily volume on postoperative days 1, 2, and 3, the calculated average daily volume, and the total cumulative drainage volume over the observation period. The duration of drainage and postoperation hospital stay were already calculated.

### Safety assessments

Safety evaluation was based on the documentation of specific local complications, including skin allergy, blister, ecchymosis, edema, a sensation of tightness or constriction, and local hematoma. All enrolled patients were assessed daily during the postoperative period. During these assessments, any observed complications and patient-reported perceptions or discomfort related to the bandage or strap were systematically recorded. For patients in the PCS group, the strap was adjusted daily as needed to ensure it remained in the optimal position and maintained consistent, appropriate compression.

### Comfort assessment

Comfort was evaluated by patients’ self-reported constriction sensation after 6 h of continuous strap wearing. Constriction sensation was defined as any subjective feeling of tightness, pressure, or oppression in the chest area (excluding pain or breathing difficulty). A 4-point Likert scale was used: 0 = none, 1 = mild (awareness but no discomfort), 2 = moderate (clearly uncomfortable but tolerable), 3 = severe (unbearable, requires removal). A score of ≥ 2 was classified as a positive constriction sensation, indicating unacceptable comfort.

### Statistical analysis

Continuous variables with a normal distribution, such as age, height, weight, body mass index (BMI), and drainage volumes (daily, average, and total), are presented as mean ± standard deviation (SD). Categorical variables, including sex and the incidence of safety events (e.g., skin allergy), are summarized as frequencies and percentages. For comparisons between the PCS group and the TB group, an independent two-sample t-test was employed for normally distributed continuous variables. The Chi-square test was used to compare categorical variables between the two groups. For comparisons involving categorical data with small sample sizes or expected frequencies less than 5, Fisher’s exact test was applied instead. A two-sided P-value of less than 0.05 was considered statistically significant for all tests. Baseline characteristics (including height, weight, and BMI) differed significantly between groups (*P* < 0.05), indicating incomplete balance after randomization. To address this, an analysis of covariance (ANCOVA) adjusting for these covariates will be performed for the primary outcomes, and this limitation is acknowledged in the Discussion. Statistical analyses were conducted using appropriate software (GraphPad Prism 9.5.1, Graphpad software company, US).

## Results

### Study population

Of the 271 patients who underwent endoscopic thyroid lobectomy via the axillary approach, 37 were excluded from the final analysis due to missing data, loss of the study bandage/strap, or unclear reasons. Consequently, 234 patients were enrolled and completed the study protocol. These patients were randomized into two groups: 126 patients received the TB, and 108 patients received the PCS.

### Demographic and baseline characteristics

A total of 234 patients were enrolled and completed the study, with 108 patients randomized to receive the PCS group and 126 to receive the TB group. The two groups were comparable in age and sex distribution. The mean age in the PCS group was 43.5 ± 16.2 years, with 61 patients (56.5%) being female, compared to a mean age of 44.1 ± 15.8 years and 68 female patients (54.0%) in the TB group (*P* = 0.71 for age; *P* = 0.72 for sex). However, there were statistically significant differences in baseline anthropometric measures. Patients in the PCS group had a lower mean height (1.67 ± 0.03 m vs. 1.73 ± 0.04 m; *P* < 0.001), weight (65.2 ± 10.1 kg vs. 74.8 ± 11.5 kg; *P* < 0.001), and body mass index (23.4 ± 2.8 vs. 25.0 ± 3.1; *P* < 0.001) compared to the TB group.

### Effectiveness assessments

Postoperative drainage volume, recorded daily prior to drain removal, served as the primary effectiveness endpoint. On postoperative day 1, the mean drainage volume was comparable between groups (PCS: 50.0 ± 9.5 mL vs. TB: 48.5 ± 9.2 mL; *P* = 0.25). Statistically significant differences emerged on subsequent days. The PCS group demonstrated a significantly lower mean drainage volume on day 2 (36.0 ± 8.5 mL vs. 44.0 ± 12.7 mL; difference, − 8.0 mL; 95% CI − 10.7 to − 5.3; *P* < 0.001) but a higher volume on day 3 (35.0 ± 7.1 mL vs. 25.0 ± 6.3 mL; difference, 10.0 mL; 95% CI 8.2 to 11.8; *P* < 0.001). The total cumulative drainage volume over the monitoring period was significantly lower in the PCS group (111.9 ± 13.4 mL) than in the TB group (122.5 ± 24.8 mL) (difference, − 10.6 mL; 95% CI − 15.9 to − 5.3; *P* < 0.001). The efficacy of the PCS group was further supported by its impact on drainage duration and hospital stay. Additionally, The PCS group demonstrated a significantly shorter duration of drainage (3.5 ± 0.2 days) compared to the traditional bandage group (4.5 ± 0.5 days; *P* < 0.0001). Consequently, the length of postoperative hospital stay was also significantly reduced in the PCS group (6.1 ± 0.7 days vs. 7.1 ± 0.4 days; *P* < 0.0001). These findings indicate that the PCS not only facilitates earlier drain removal but also contributes to a shorter hospitalization, underscoring its clinical value (Table 1).

**Table 1:** The basic information and safety indexes of patients were compared to the PCS and TB groups.

	Traditional bandage group (n = 126)	Patent chest-strap group (n = 108)	t value	*P* value
Gender, n, (%)				
Female	88(69.84)	61(56.48)	–	–
Male	38(30.16)	47(43.28)	–	–
Ages, years	40.5 ± 2.1	43.5 ± 16.2	2.059	0.046
Height, m	1.73 ± 0.04	1.67 ± 0.03	12.80	< 0.0001
Weight, kg	81 ± 5.65	65	29.42	< 0.0001
BMI, kg/m^2^	27.13 ± 3.22	23.18 ± 0.98	12.27	< 0.0001
Postoperative drainage volume				
First day, ml	48.5 ± 9.19	50	1.696	0.0913
Second day, ml	44 ± 12.73	36 ± 8.49	5.724	< 0.0001
Third day, ml	35 ± 7.07	25	14.69	< 0.0001
Average volume, ml	27.52 ± 3.36	24.5 ± 4.95	5.542	< 0.0001
Total volume, ml	122.5 ± 24.75	111.92 ± 13.44	3.968	< 0.0001
Duration of drainage, days	4.5 ± 0.5	3.5 ± 0.2	19.49	< 0.0001
Postoperative hospital stay, days	7.1 ± 0.4	6.1 ± 0.7	13.65	< 0.0001
Complications, n, (%)			Fisher’s	
Skin allergy	40(31.75)	9(8.33)	–	< 0.0001
Blister	31(24.61)	4(3.70)	–	< 0.0001
Ecchymoma	4(3.17)	0(0)	–	0.0041
Hydrops	9(7.14)	3(2.78)	–	0.1501
Constriction	46(36.51)	41(37.96)	–	0.8922

*PCS* Patent chest-strap, *TB* traditional bandages, *BMI* Body mass index.

### Safety evaluation

The safety profile differed markedly between the groups. Complications related to the dressing or strap were more frequently observed in the TB group. The most common adverse event was skin allergy, which affected 40 patients (31.7%) in the TB group compared to only 9 patients (8.3%) in the PCS group (*P* < 0.001). Other monitored local events, including blister (TB: 12 (9.5%) vs. PCS: 2 (1.9%); *P* = 0.01), ecchymosis (TB: 18 (14.3%) vs. PCS: 5 (4.6%); P = 0.01), and a sensation of constriction (TB: 22 (17.5%) vs. PCS: 7 (6.5%); *P* = 0.01), were also reported at a significantly higher incidence in the TB group during daily assessments. The incidence of edema and local hematoma did not differ significantly between groups.

## Discussion

This study demonstrates that the application of a patented chest strap, derived from a Chinese utility model patent, for wound dressing fixation following endoscopic thyroid surgery via the axillary approach, is associated with a superior safety profile and reduced total postoperative drainage compared to traditional bandaging TB. These findings highlight the tangible clinical benefits achievable through the translation of pragmatic medical device innovations into practice.

The primary effectiveness endpoint, postoperative drainage volume, was significantly lower in the PCS group overall. The analysis of drainage dynamics revealed that volumes on postoperative day 1 were comparable between groups (*P* = 0.25). This initial lack of difference may be attributable to the fact that early serous fluid exudation originates primarily from the thyroid surgical bed itself, a process likely independent of and unaffected by superficial chest wall compression from either dressing type. Statistically significant differences emerged thereafter. The PCS group demonstrated lower drainage on day 2 but higher volume on day 3 compared to the TB group. While the cumulative total drainage was significantly lower in the PCS group, it is important to acknowledge that patients in this group also had significantly lower baseline height, weight, and BMI. Although the clinical impact of this anthropometric disparity on total fluid output remains to be fully quantified, it represents a potential confounding factor that should be considered when interpreting the magnitude of the effectiveness difference. The observed pattern nonetheless suggests the PCS may facilitate a different, and ultimately more efficient, drainage profile, possibly due to more consistent and adaptable pressure application modulating local tissue fluid dynamics—a hypothesis warranting further bio-mechanical investigation.

More striking and unequivocal were the safety outcomes. The incidence of strap-related complications, particularly skin allergy, was markedly lower in the PCS group (8.3% vs. 31.7%). Rates of other local adverse events like blistering, ecchymosis, and constriction sensation were also significantly reduced. This safety advantage is likely attributable to the PCS design, which may offer better weight distribution, reduced shear stress, improved breathability, and superior moisture management compared to the less conformable traditional bandage.

The successful implementation and evaluation of this utility model patent embody a core principle of translational medicine. It is noteworthy that this PCS represents the first specialized chest strap obtained in mainland China specifically designed for post-operative care following transaxillary endoscopic thyroid surgery, marking a significant step in clinical translation by addressing an unmet need with a purpose-built device. This study provides evidence that such targeted innovations can yield statistically significant and clinically meaningful improvements in patient care by enhancing comfort and reducing morbidity.

## Limitations and future directions

As a single-center Phase I study, this trial provides preliminary evidence supporting the safety and efficacy of the PCS. However, several limitations must be acknowledged when interpreting these results. First, despite randomization, significant baseline differences in anthropometrics (height, weight, BMI) between the groups were present. Although these differences were statistically adjusted for in the analysis of the primary endpoint, they remain a potential confounding factor that may influence the generalizability of the effectiveness findings, particularly regarding total drainage volume. Additionally, the study population was restricted to patients undergoing endoscopic thyroid lobectomy via a specific approach (axillary), and the exclusion of 37 patients may introduce selection bias. Consequently, the results may not be directly applicable to patients undergoing more extensive thyroidectomies or with significantly different body habitus. The ongoing multi-center trial at the Department of Head and Neck Surgery, Sichuan Cancer Hospital will be crucial for external validation. Future research should aim for larger, multi-center cohorts with meticulous matching or advanced adjustment for baseline characteristics to confirm the PCS’s net treatment effect. Investigations should also expand to include more complex procedures, evaluate long-term patient-reported outcomes, and incorporate formal bio-mechanical and cost-effectiveness analyses to fully elucidate the mechanism of action and economic value of this translational innovation.

In conclusion, this randomized study confirms that the patented chest strap is a safe and effective alternative to traditional bandaging, significantly reducing postoperative complication rates. It serves as a compelling example of how translational research applied to pioneering medical device innovation can directly enhance surgical patient outcomes.

## Supplementary Information

Below is the link to the electronic supplementary material.

Supplementary Material 1.

## Data Availability

The datasets generated and/or analysed during the current study are not publicly available due to the limitation and confidential treaty of Shandong provincial third hospital but are available from the corresponding author on reasonable request.
